# Contribution of porcine aminopeptidase N to porcine deltacoronavirus infection

**DOI:** 10.1038/s41426-018-0068-3

**Published:** 2018-04-11

**Authors:** Xinyu Zhu, Shudan Liu, Xunlei Wang, Zhaochen Luo, Yuejun Shi, Dang Wang, Guiqing Peng, Huanchun Chen, Liurong Fang, Shaobo Xiao

**Affiliations:** 1 State Key Laboratory of Agricultural Microbiology, College of Veterinary Medicine, Huazhong Agricultural University, Wuhan, 430070 China; 2 Key Laboratory of Preventive Veterinary Medicine in Hubei Province, The Cooperative Innovation Center for Sustainable Pig Production, Wuhan, 430070 China

## Abstract

Porcine deltacoronavirus (PDCoV), a member of genus *Deltacoronavirus*, is an emerging swine enteropathogenic coronavirus (CoV). Although outstanding efforts have led to the identification of *Alphacoronavirus* and *Betacoronavirus* receptors, the receptor for *Deltacoronavirus* is unclear. Here, we compared the amino acid sequences of several representative CoVs. Phylogenetic analysis showed that PDCoV spike (S) protein was close to the cluster containing transmissible gastroenteritis virus (TGEV), which utilizes porcine aminopeptidase N (pAPN) as a functional receptor. Ectopic expression of pAPN in non-susceptible BHK-21 cells rendered them susceptible to PDCoV. These results indicate that pAPN may be a functional receptor for PDCoV infection. However, treatment with APN-specific antibody and inhibitors did not completely block PDCoV infection in IPI-2I porcine intestinal epithelial cells. pAPN knockout in IPI-2I cells completely blocked TGEV infection but only slightly decreased PDCoV infection. Homologous modeling of pAPN with the S1 C-terminal domain (S1-CTD) of PDCoV or TGEV showed that TGEV S1-CTD adopted β-turns (β1–β2 and β3–β4), forming the tip of a β-barrel, to recognize pAPN. However, only the top residues in the β1–β2 turn of PDCoV S1-CTD had the possibility to support an interaction with pAPN, and the β3–β4 turn failed to contact pAPN. We also discuss the evolution and variation of PDCoV S1-CTD based on structure information, providing clues to explain the usage of pAPN by PDCoV. Taken together, the results presented herein reveal that pAPN is likely not a critical functional receptor for PDCoV, although it is involved in PDCoV infection.

## Introduction

Porcine deltacoronavirus (PDCoV) is an emerging swine enteropathogenic coronavirus (CoV) belonging to the genus *Deltacoronavirus* of the family *Coronaviridae* within the order *Nidovirales*^1–4^. Like other CoVs, PDCoV is an enveloped virus that contains positive, single-stranded genomic RNA^5, 6^. PDCoV was first identified in 2012 during molecular surveillance of CoVs in mammals and birds in Hong Kong^6^. The first PDCoV outbreak was reported in 2014 in the United States^7^, causing severe diarrhea, vomiting, and mortality in piglets^3, 4, 8, 9^. Thereafter, PDCoV was also detected in China^10–13^, Canada, South Korea^14, 15^, Lao People’s Democratic Republic, Thailand^16^, and Vietnam^17, 18^, gaining considerable attention^19–21^.

In general, CoVs have a limited host range and tissue tropism. The interaction between CoV spike (S) proteins and specific cellular receptors on host cell surfaces mediates viral attachment and fusion of viral and cellular membranes, playing a vital role in successful infection in the host^22–24^. The CoV S protein is a type I transmembrane glycoprotein with high molecular weight that protrudes from the surface of virions. The amino-terminal S1 domain is responsible for the recognition of cellular receptors, and the carboxy-terminal S2 domain mediates the subsequent membrane fusion process^25^. Recently, two research groups independently resolved the structure of PDCoV S protein by cryo-electron microscopy^26, 27^. PDCoV S protein is a trimer containing three receptor-binding S1 subunits and membrane-fusion S2 subunits^27^. The C-terminal domain (CTD) of the PDCoV S1 subunit that is responsible for receptor binding shares a similar structural fold with alphacoronavirus.

To date, a series of cellular receptors for different genera of CoVs have been identified. For example, aminopeptidase N (APN, also called CD13) is the functional receptor for human coronavirus 229E (HCoV-229E)^28^, feline infectious peritonitis virus^29^, canine CoV^30^, and transmissible gastroenteritis virus (TGEV)^31^. Angiotensin-converting enzyme 2 is utilized by HCoV-NL63 and severe acute respiratory syndrome coronavirus (SARS-CoV)^24, 32^. Middle East respiratory syndrome coronavirus (MERS-CoV) S protein employs dipeptidyl peptidase 4 (also called CD26) as its receptor^33^. Carcinoembryonic antigen-related cell adhesion molecule 1 is reported to mediate viral infection by interacting with mouse hepatitis virus (MHV) S protein^34^. However, the relationship between hosts and members of genus *Deltacoronavirus* remains unknown.

In this study, we investigated the role of porcine APN (pAPN) in PDCoV infection. We found that ectopic expression of pAPN rendered non-susceptible cells susceptible to PDCoV infection and promoted PDCoV infection in poorly susceptible cells. However, pAPN knockout or treatment with APN-specific antibody and inhibitor only decreased PDCoV infection to some degree. Additionally, pAPN knockout in porcine intestinal epithelial (IPI-2I) cells, a cell line established from porcine ileum, did not completely block PDCoV infection but significantly affected viral replication. We also demonstrated that the APN enzymatic activity inhibitor did not disrupt PDCoV infection, indicating pAPN enzymatic activity is not involved in this process. Our work suggests pAPN is not a critical receptor but is an important factor during PDCoV infection.

## Results

### Phylogenetic analysis of PDCoV S protein

**Fig. 1:**
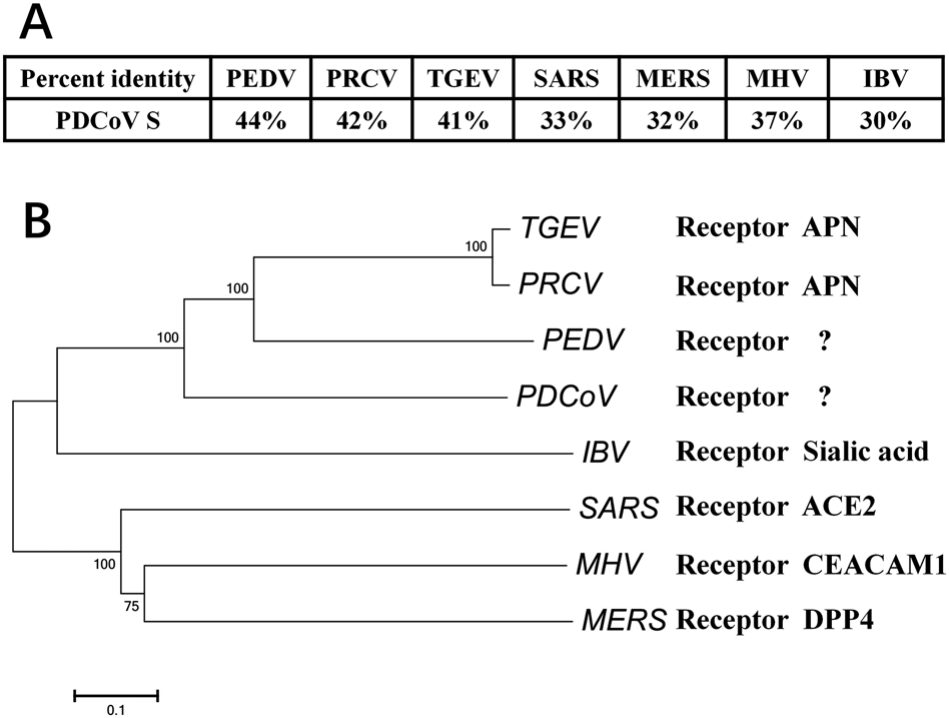
Amino acid sequence alignments and phylogenetic relationships of S protein from different CoVs. **a** Alignment of the deduced amino acid sequence of S proteins from PDCoV (GenBank accession no. ALS54086.1), PRCV (GenBank accession no. ABG89317.1), TGEV (GenBank accession no. ADY39740.1), PEDV (GenBank accession no. AHZ94887.1), SARS-CoV (GenBank accession no. ABD73002.1), MERS-CoV (GenBank accession no. AKS48062.1), MHV (GenBank accession no. AFD97607.1), and IBV (GenBank accession no. AKN20490.1). **b** S protein sequences from different CoVs were analyzed with the neighbor-joining method using Molecular Evolutionary Genetics Analysis (MEGA) software^58^. Each bootstrap value was determined by 1000 replicates, and bootstrap values >50% are shown. The scale bar represents the relationship between line lengths and sequence dissimilarities

Because of the dominant role of CoV S protein in receptor recognition and viral entry, we conducted amino acid sequence alignments of PDCoV S proteins, together with S proteins from three swine CoVs (TGEV; porcine epidemic diarrhea virus, PEDV; and porcine respiratory coronavirus, PRCV) and four representative CoVs from different genera (*Betacoronavirus* SARS-CoV, MERS-CoV, and MHV; *Gammacoronavirus* infectious bronchitis virus (IBV)). PDCoV S displayed higher homology with PEDV, PRCV, and TGEV (Fig. 1a). pAPN acts as a functional cellular receptor for TGEV and PRCV infection^31, 35^. Whether pAPN is indeed a functional receptor for PEDV remains controversial, but it is involved in PEDV infection^36–38^. Phylogenetic analysis indicated that PDCoV S is close to the cluster containing PEDV, TGEV, and PRCV (Fig. 1b). Thus, we evaluated the role of pAPN in PDCoV infection.

### Non-susceptible cells expressing pAPN are susceptible to PDCoV

**Fig. 2:**
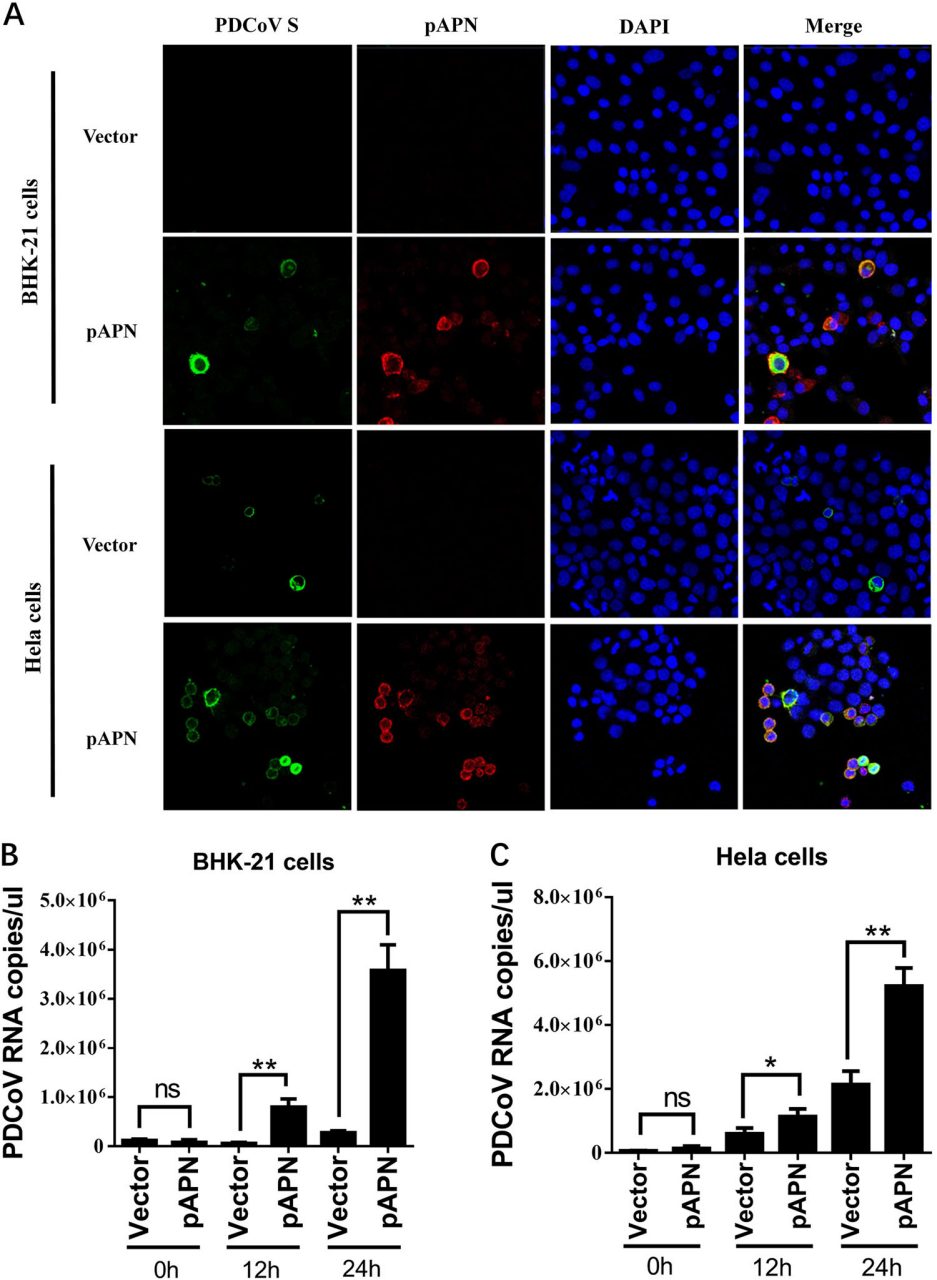
pAPN overexpression promotes PDCoV infection. **a** BHK-21 cells or HeLa cells were cultured in 24-well plates and transfected with 1 μg of pAPN expression plasmid or emptor vector. After 24 h, cells were infected with PDCoV (MOI = 2). At 24 h post infection, cells were fixed and analyzed for IFA. Mouse monoclonal antibody against PDCoV S was used to detect PDCoV-infected cells (green). The anti-Flag rabbit polyclonal antibody was used to stain for ectopic expression of APN protein (red). DAPI was applied to detect nuclei (blue). **b**, **c** BHK-21 cells (**b**) or HeLa cells (**c**) were transfected as described in **a** and then infected with PDCoV (MOI = 2). At different time points (0, 12, 24 h) post infection, cells were collected for RT-qPCR with primers targeting the PDCoV nsp16 gene to measure viral genome copies. Data are expressed as the mean ± SD for triplicate wells. Statistical significance was determined by Student’s *t* test; ns, *P* > 0.05; *P < 0.05; **P < 0.01

To determine whether pAPN contributes to PDCoV infection, we explored whether ectopic expression of pAPN in non-susceptible cells can cause PDCoV infection. Primary experiments showed that baby hamster kidney (BHK)-21 cells were non-susceptible and HeLa cells were slightly susceptible to PDCoV infection. pAPN overexpression in these cells was induced by transient transfection of the pAPN expression plasmid. PDCoV S-specific fluorescence was observed in BHK-21 cells transfected with the pAPN expression plasmid, but not in cells transfected with control vector (Fig. 2a). Moreover, HeLa cells showed greater infection by PDCoV after transfection with pAPN expression plasmid compared with cells transfected with control vector (Fig. 2a).

To further investigate whether pAPN-mediated infection in transfected BHK-21 and HeLa cells is productive, quantitative real-time PCR (RT-qPCR) was performed to detect the PDCoV genome RNA copies at different time points post infection. In BHK-21 cells, ectopic expression of pAPN clearly promoted increased viral genome RNA copies compared with cells transfected with control vector. Moreover, viral genome RNA copies increased over time, confirming that a productive PDCoV infection was established in BHK-21 cells with the help of pAPN (Fig. 2b). Although the amount of genome RNA showed a slight increase in empty vector-transfected HeLa cells at 12 h and 24 h post infection, pAPN-transfected HeLa cells showed a stronger ability to promote PDCoV replication compared with control cells (Fig. 2c). These results strongly suggest that HeLa cells and BHK-21 cells expressing pAPN are equipped to support PDCoV infection, implying that pAPN does serve as an important factor for PDCoV infection.

### Treatment with polyclonal APN antibody inhibits PDCoV infection

**Fig. 3:**
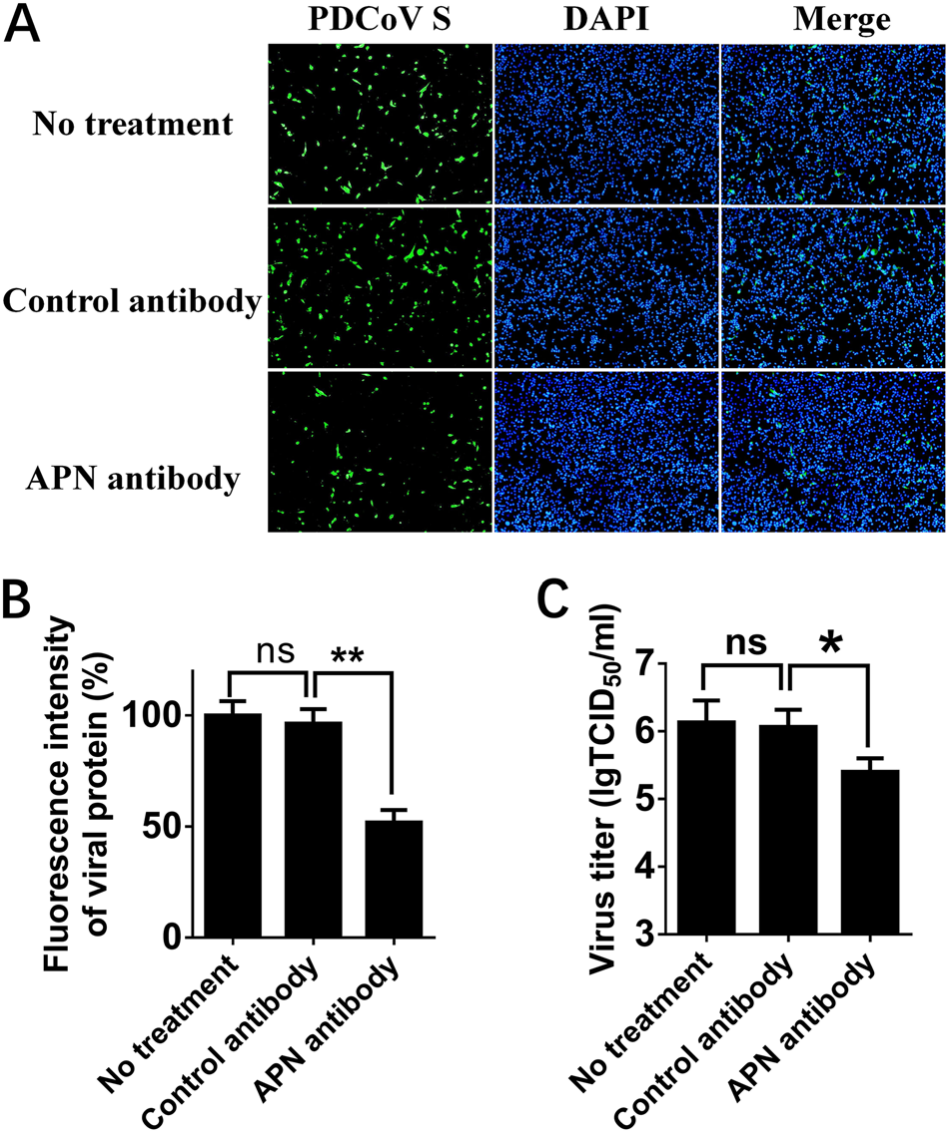
Effects of APN-specific antibody on PDCoV infection. **a** IPI-2I cells were treated with anti-APN rabbit polyclonal antibody, anti-Flag rabbit polyclonal antibody or no antibody (control) for 2 h and then infected with PDCoV (MOI = 2). After 24 h, cells were analyzed by IFA. Mouse monoclonal antibody against PDCoV S was used to detect PDCoV-infected cells (green). DAPI was applied to detect nuclei (blue). **b** The fluorescence intensity in **a** was quantified with the software ImageJ. Error bars show standard deviations. **c** IPI-2I cells were treated with antibody and infected with PDCoV as described in **a**. Cells underwent three freeze/thaw cycles, and LLC-PK1 cells were used to measure viral titer by TCID_50_ assay. Data are expressed as the mean ± SD for triplicate samples. Statistical significance was determined by Student’s *t* test; *P < 0.05

In previous studies of various CoVs, pretreatment with antibody against receptor neutralized viral infectivity^32, 33^. To determine the effect of pAPN in PDCoV infection, we first examined whether polyclonal APN antibody can affect PDCoV infection in susceptible cells. Porcine IPI-2I cells, established from the ileum of an adult boar, are highly susceptible to PDCoV infection. Thus, we compared PDCoV infectivity in IPI-2I cells treated with APN-specific antibody, control antibody and no antibody. The results of immunofluorescence assay (IFA) showed that PDCoV S-specific fluorescence was reduced in cells treated with APN antibody (Fig. 3a, b), indicating that treatment with APN-specific antibody reduced PDCoV infection. The viral titer (indicated by TCID_50_) was also decreased with APN-specific antibody treatment (Fig. 3c).

### pAPN enzymatic activity is not involved in PDCoV infection

**Fig. 4:**
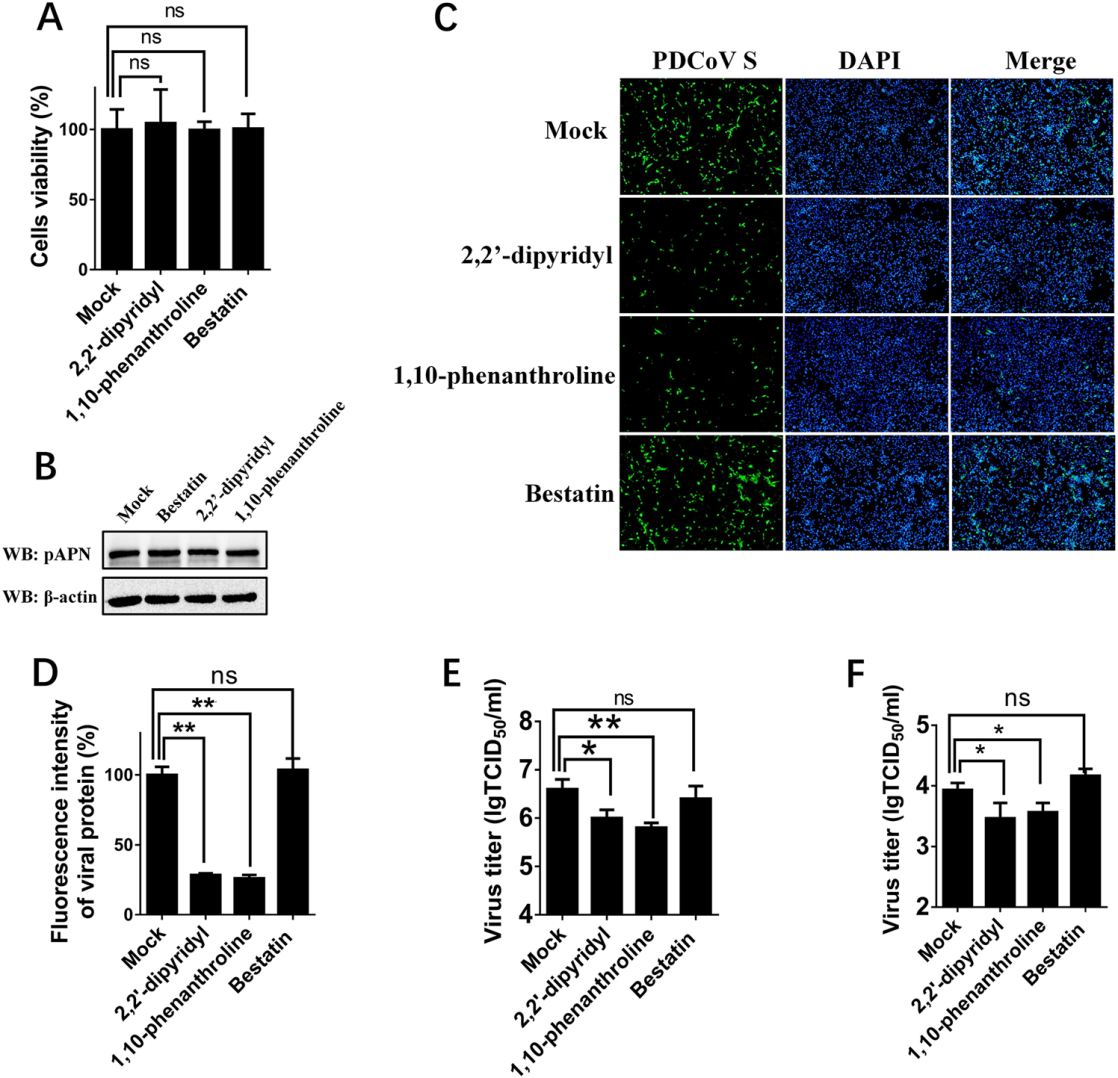
Effects of APN inhibitors on PDCoV infection. **a** Cytotoxicity detection of 2,2′-dipyridyl, 1,10-phenanthroline, and bestatin by MTT assay. IPI-2I cells cultured in the 96-well plates were incubated with 2,2′-dipyridyl (250 μM), 1,10-phenanthroline (15 μM), or bestatin (300 μM). At 24 h after incubation, the inhibitors were removed, and MTT reagents (20 μL, 5 mg/mL) were added. After another 4 h incubation, the medium was discarded, and 150 μL of dimethyl sulfoxide (DMSO) solution was added. The OD value at 570 nm was measured. **b** The expression levels of APN in cells after treatment with the three inhibitors. IPI-2I cells were cultured in six-well plates and treated with 2,2′-dipyridyl (250 μM), 1,10-phenanthroline (15 μM), or bestatin (300 μM). At 24 h after treatment, the expression of endogenous pAPN was detected by western blot with anti-APN rabbit polyclonal antibody. **c** 2,2′-Dipyridyl (250 μM), 1,10-phenanthroline (15 μM), or bestatin (300 μM) was added to IPI-2I cells for 1 h. Cells were then infected with PDCoV (MOI = 2). At 24 h post infection, cells were analyzed by IFA. Mouse monoclonal antibody against PDCoV S was used to detect PDCoV-infected cells (green). DAPI was applied to detect nuclei (blue). **d** The fluorescence intensity in **c** was quantified with ImageJ. **e**, **f** IPI-2I cells were treated with the three inhibitors as described in **c** and infected with PDCoV (MOI = 2 in **e** or MOI = 0.2 in **f**). At 24 h post infection, cells were collected, and the TCID_50_ was determined in LLC-PK1 cells. Data are expressed as the mean ± SD for triplicate samples. Statistical significance was determined by Student’s *t* test; ns, *P* > 0.05; *P < 0.05; **P < 0.01

APN is a zinc-dependent metalloprotease, and previous studies suggested that its enzymatic activity or epitope independent of its enzymatic activity are associated with APN function as a viral receptor^37, 39^. Thus, three APN-specific inhibitors, bestatin (a small inhibitory molecule that competitively binds to the catalytic site of APN), 2,2′-dipyridyl, and 1,10-phenanthroline (a zinc-chelating molecule that impairs APN epitope conformation)^36^, were used to determine the role of APN in PDCoV infection. First, MTT assays and western blots were performed to detect the cytotoxicity of the three inhibitors and whether they affected the expression of endogenous APN in IPI-2I cells. Nearly no cytotoxicity could be detected (Fig. 4a), and APN expression was not affected in cells treated with any of the three inhibitors (Fig. 4b). Then, IFA and TCID_50_ assays were performed to analyze the roles of three inhibitors in PDCoV infection. The results of IFA showed that the numbers of PDCoV S-specific fluorescence-positive cells were reduced in 2,2′-dipyridyl- and 1,10-phenanthroline-treated cells, but not in bestatin-treated cells, compared with control cells (Fig. 4c, d). The viral titers after inhibitor treatments showed similar results under infection with PDCoV at different MOIs [MOI = 2 and MOI = 0.2] (Fig. 4e, f), suggesting that pAPN’s epitope conformation, not its enzymatic activity, is involved in PDCoV infection.

### pAPN knockout in IPI-2I cells decreases PDCoV infection

**Fig. 5:**
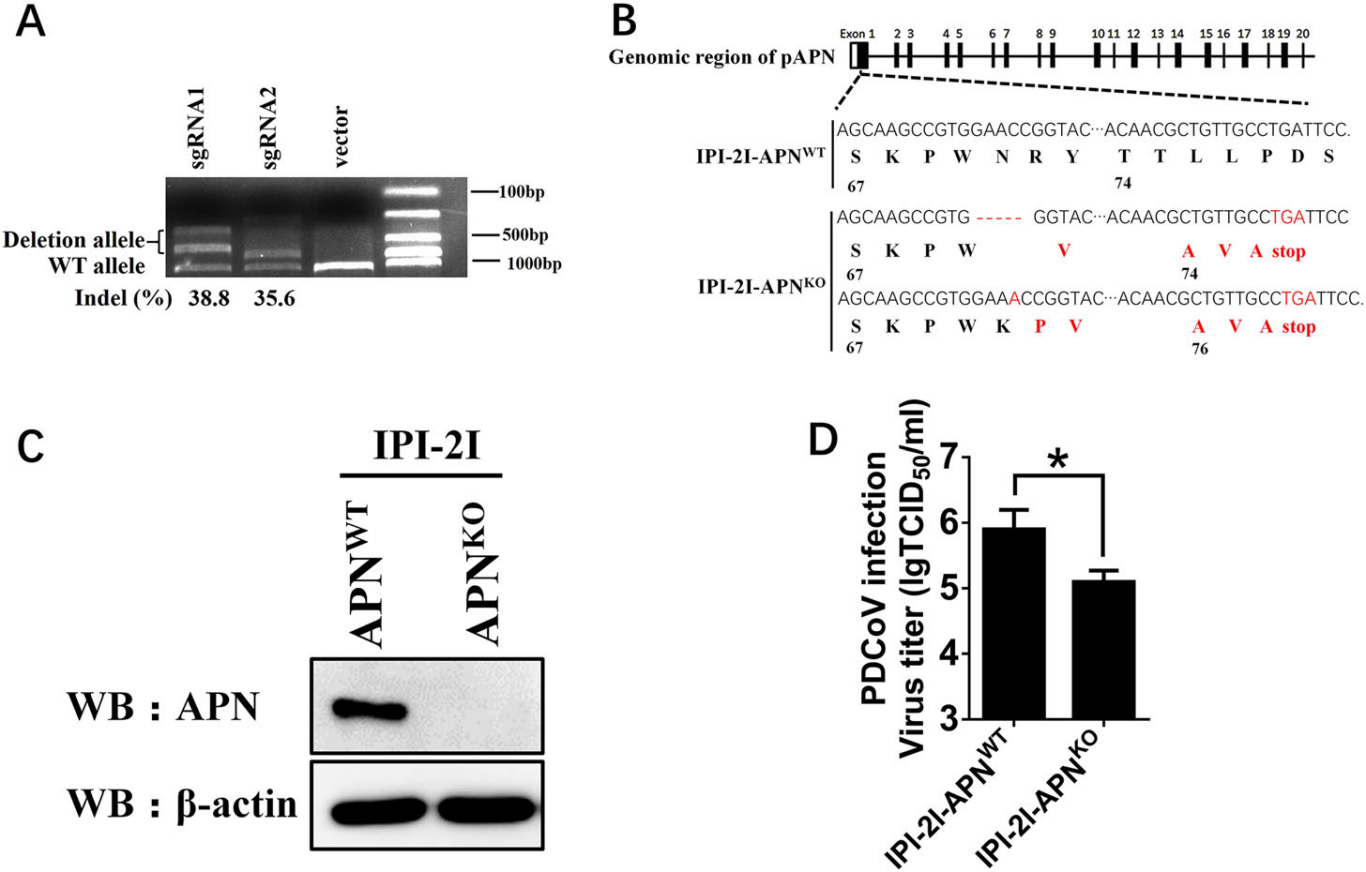
pAPN knockout in IPI-2I cells inhibits PDCoV infection. **a** sgRNA1 and sgRNA2 expression plasmids (1 μg) targeting exon 1 of the pAPN genome were transfected into IPI-2I cells. Total DNA was collected, and the surveyor nuclease assay was conducted to calculate indel occurrence. **b** The target sequence in isolated clonal cells was amplified through PCR and cloned into the pMD18-T vector. Edited nucleotides in pAPN gene alleles are shown according to sequencing analysis. **c** Isolated clonal IPI-2I-APN^KO^ cells were cultured in 6-well plates, and the expression of endogenous pAPN was detected by western blot with anti-APN rabbit polyclonal antibody. **d** IPI-2I-APN^KO^ and IPI-2I-APN^WT^ cells were cultured in 24-well plates and infected with PDCoV (MOI = 2). At 24 h post infection, cells were collected, and the viral titer was determined by TCID_50_ assay in LLC-PK1 cells. Data are expressed as the mean ± SD for triplicate samples. Statistical significance was determined by Student’s *t* test; *P < 0.05

To determine whether pAPN is essential to PDCoV infection, the CRISPR/Cas9 system was applied to establish pAPN knockout cell lines. Two sgRNAs were designed to target exon 1 of pAPN in IPI-2I cells. The surveyor nuclease assay indicated that the indel occurrence rates of the target nucleotide caused by the two sgRNAs were 38.8% and 35.6%, respectively (Fig. 5a). Isolated pAPN knockout IPI-2I (IPI-2I-APN^KO^) cell lines were further confirmed by sequencing and western blot analyses (Fig. 5b, c). Sequencing analysis demonstrated the alleles of the APN gene in the clonal cell line harbored a 5-nt deletion and a 1-nt insertion in exon 1 of the APN genome, resulting in the production of a truncated peptide with no function (Fig. 5b). Therefore, IPI-2I-APN^KO^ cell lines were used to functionally assess the role of endogenous pAPN in PDCoV infection. Compared with wild-type IPI-2I cells (IPI-2I-APN^WT^), the viral titer in IPI-2I-APN^KO^ cell lines was decreased by 1 log (Fig. 5d). We tested another isolated pAPN knockout cell line, IPI-2I-APN^KO2^, and similar results were observed (data not shown). These results demonstrate that knockout of APN expression in IPI-2I cells decreased PDCoV infection.

### pAPN affects an early step of PDCoV entry rather than viral assembly or release in IPI-2I cells

**Fig. 6:**
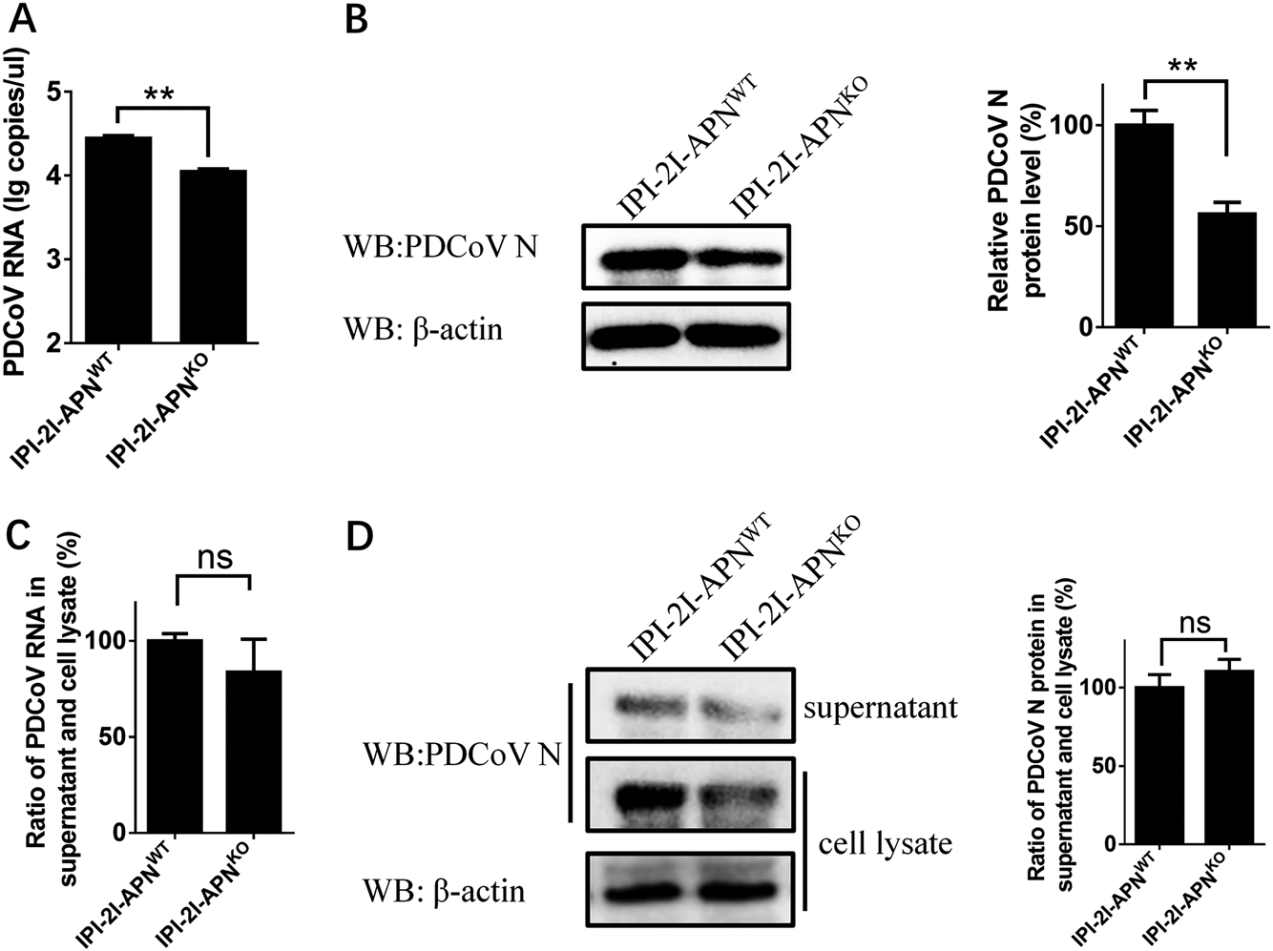
pAPN is involved in an early step of PDCoV entry rather than viral assembly or release. **a**, **b** IPI-2I-APN^WT^ and IPI-2I-APN^KO^ cells were inoculated with PDCoV (MOI = 30) at 4 °C. After 1 h, the infected cells were washed three times with cold PBS and cultured at 37 °C for another 1 h. Then, the infected cells were washed with citrate buffer solution (pH = 3) to remove the bound but non-internalized virus particles. The cells were harvested for RT-qPCR assay (**a**) and western blot (**b**). **c**, **d** IPI-2I-APN^WT^ and IPI-2I-APN^KO^ cells were inoculated with PDCoV (MOI = 2) at 4 °C for 1 h and washed with cold PBS. At 24 h post infection, the ratio of PDCoV RNA copy number and PDCoV N protein level in the supernatants vs. the cell lysates were separately detected by RT-qPCR (**c**) and western blot (**d**), respectively. The PDCoV protein level was quantified by ImageJ. Statistical significance was determined by Student’s *t* test; ns, *P* > 0.05; *P < 0.05; **P < 0.01

Because pAPN knockout in IPI-2I cells decreased PDCoV infection, we further investigated which step is affected by APN in the replication cycle of PDCoV infection. Considering the cell membrane localization of APN, pAPN in IPI-2I cells is most likely to affect the step of viral adsorption, invasion or release, each of which occurs on the cell membrane. First, the internalization assay was conducted to analyze the earliest viral entry process. RT-qPCR and western blot showed that viral genome RNA copies and protein levels in IPI-2I-APN^KO^ cells were both lower than those in IPI-2I-APN^WT^ cells (Fig. 6a, b). To further detect the viral assembly or release, IPI-2I-APN^KO^ and IPI-2I-APN^WT^ cell lines were infected with PDCoV, respectively. At 24 h after infection, the cells and supernatants were separately collected for RT-qPCR and western blot. The ratios of the viral genome RNA copies and protein levels were almost identical between supernatant and cell lysates (Fig. 6c, d), suggesting that pAPN has no effect on viral assembly or release. Taken together, these results indicate that pAPN mainly affects an early step of PDCoV entry in IPI-2I cells.

### Knockout of pAPN gene in IPI-2I cell lines completely blocks TGEV infection but only slightly decreases PDCoV infection

**Fig. 7:**
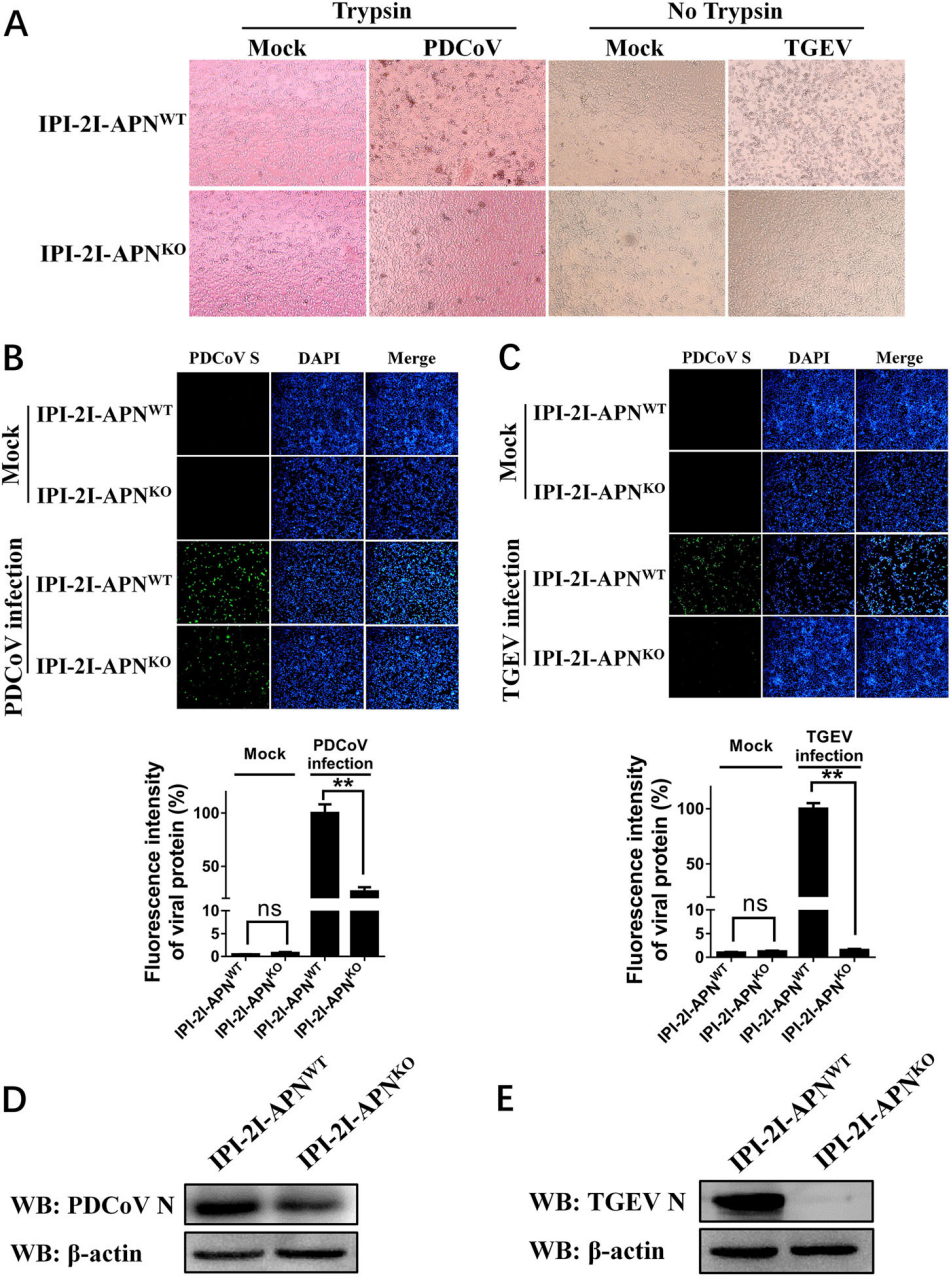
Comparison of PDCoV and TGEV infection in IPI-2I-APN^KO^ cells. **a** IPI-2I-APN^WT^ and IPI-2I-APN^KO^ cells seeded in 24-well plates were inoculated with PDCoV (MOI = 2) or TGEV (MOI = 1). At 24 h post infection, CPE was examined to compare the production of infectious progeny virus. **b**, **c** IPI-2I-APN^WT^ and IPI-2I-APN^KO^ cells were infected with PDCoV (**b**) or TGEV (**c**) as described in **a**. At 24 h post infection, PDCoV S- or TGEV M-specific fluorescence was detected by IFA. The fluorescence intensity was quantified with ImageJ. **d**, **e** IPI-2I-APN^WT^ and IPI-2I-APN^KO^ cells were seeded in six-well plates and infected with PDCoV (**d**) or TGEV (**e**) as described in **a**. At 24 h post infection, cells were collected for western blot analysis with antibodies against N protein of PDCoV (**d**) or TGEV (**e**)

pAPN is reported to act as a critical cellular receptor during TGEV entry^31^. In the light of the limited effect of pAPN in PDCoV infection, we compared the difference between PDCoV and TGEV infection in IPI-2I cells to better understand the role of pAPN. IPI-2I-APN^WT^ and IPI-2I-APN^KO^ cell lines were incubated with the same dose of TGEV or PDCoV. Cytopathic effects (CPEs) caused by PDCoV infection were observed in both IPI-2I-APN^WT^ and IPI-2I-APN^KO^ cells, even though IPI-2I-APN^KO^ cells presented a mild CPE (Fig. 7a). However, loss of the APN gene in IPI-2I-APN^KO^ cells led to the disappearance of CPEs in TGEV infection (Fig. 7a). PDCoV S protein and TGEV M protein levels in IPI-2I-APN^WT^ and IPI-2I-APN^KO^ cells were also analyzed by IFAs. As expected, PDCoV S-specific fluorescence in IPI-2I-APN^KO^ cells was partially reduced (Fig. 7b), while TGEV M-specific fluorescence was undetectable (Fig. 7c). Similar results were demonstrated by western blot using antibodies against viral N proteins (Fig. 7d, e). These results indicate that pAPN knockout in IPI-2I cells absolutely blocked TGEV infection and only partly decreased PDCoV infection.

### Homologous modeling of pAPN with S1-CTD of PDCoV or TGEV

**Fig. 8:**
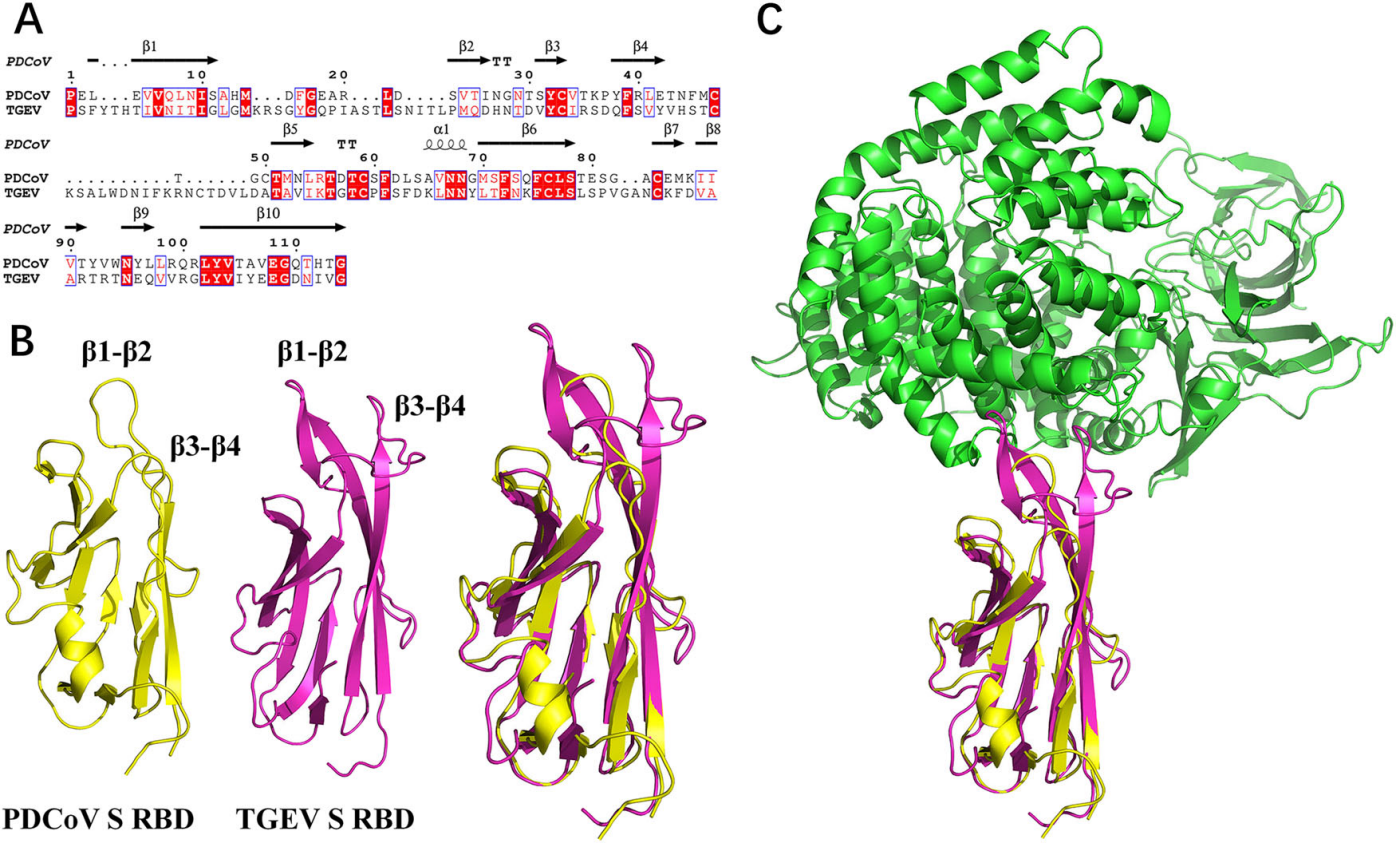
Homologous modeling of PDCoV or TGEV S1-CTD with pAPN. **a** Sequence alignment was conducted with PDCoV S1-CTD (GenBank accession no. ALS54086.1) and TGEV S1-CTD (GenBank accession no. ADY39740.1). The secondary structures of S1-CTD were analyzed using the ESPript website (http://espript.ibcp.fr/ESPript/ESPript/index.php). **b**, **c** The three-dimensional structures of PDCoV S1-CTD (yellow; PDB ID 6B7N), TGEV S1-CTD (red; PDB ID 4F2M), and pAPN (blue; PDB ID 4F5C) obtained from the Protein Data Bank were analyzed with PyMOL software

To further explain the different roles of pAPN in PDCoV and TGEV, we compared the amino acid sequences of TGEV and PDCoV S1-CTD. Although the two proteins shared similar secondary structure elements, the N-terminus of PDCoV S1-CTD was shorter than that of TGEV, which led to a shorter turn of β1–β2 and of β3–β4 in the PDCoV structure (Fig. 8a, b). Hence, the homologous model showed that S1-CTD of both PDCoV and TGEV adopted β-turns (β1–β2 and β3–β4), forming the tip of the β-barrel to recognize pAPN (Fig. 8c). For TGEV, residues in the two β-turns both contacted pAPN comfortably, forming a contact network (Fig. 8c). Conversely, only the top residues in the β1–β2 turn of PDCoV supported an interaction with pAPN, and the β3–β4 turn failed to contact pAPN (Fig. 8c), indicating that its contribution to the interaction with pAPN is smaller than that of TGEV. Overall, the different lengths of the two β-turns (β1–β2 and β3–β4) between PDCoV and TGEV may determine their variability in pAPN usage during viral entry.

## Discussion

As an emerging swine enteropathogenic CoV and the sole member of the genus *Deltacoronavirus* that has been successfully isolated by cell culture in vitro, PDCoV is a good model to study deltacoronaviruses. However, very little is known about this emerging virus, including its receptor and infection mechanisms. A recent study showed that the β-sandwich core structure of PDCoV S1-CTD, which may be responsible for receptor recognition, is similar to that of alphacoronaviruses, which mainly use APN as a cellular receptor^27^. Furthermore, PDCoV in infected pigs was primarily detected in the villous epithelium of the mid-jejunum to ileum^20^. Large amounts of APN are also expressed on the surface of porcine small intestine enterocytes^31^. Thus, in this study, we investigated the role of pAPN in PDCoV infection. Interestingly, ectopic expression of pAPN successfully reestablished PDCoV infection in non-susceptible BHK-21 cells, and pAPN overexpression effectively promoted PDCoV infection in poorly susceptible HeLa cells expressing pAPN. However, pAPN knockout in IPI-2I cells, a cell line that was established from porcine ileum and is highly susceptible to PDCoV infection, did not completely block PDCoV infection. We also performed pAPN knock-down experiments with pAPN-specific siRNA in LLC-PK1 (porcine kidney) cells, and the viral titers were decreased by only 0.6 log in pAPN-knock-down LLC-PK1 cells compared to the LLC-PK1 cells transfected with control siRNA (data not shown). A recent study found no interaction between purified pAPN and PDCoV S1-CTD by dot-blot assay^27^. Although we detected a possible weak interaction with the purified full-length PDCoV S protein and pAPN through molecular sieve analysis, a direct interaction could not be confirmed by pull-down assay (data not shown). In contrast, another CoV, TGEV, which uses pAPN as a functional receptor, failed to infect IPI-2I-APN^KO^ cells, as demonstrated by CPE, viral protein expression, and viral genome replication. These results indicate that pAPN plays a decisive role in TGEV infection and only a contributing role in PDCoV infection in IPI-2I cells. Coincidentally, pAPN was initially identified as a PEDV-binding protein in swine kidney cells by virus overlay protein binding assay, and non-susceptible cell lines expressing pAPN became susceptible to PEDV^40, 41^. However, more recent evidence showed that pAPN knockout did not decrease PEDV infection, and no interaction between PEDV S1 and pAPN was detected, implying that pAPN is not a functional receptor for PEDV entry^37^. Thus, whether pAPN promotes PDCoV or PEDV infection through direct interaction with S protein or along with other cellular proteins requires further investigation.

APN is a 150-kDa type II glycoprotein in the metalloprotease family^31^. As a zinc-dependent metalloprotease, APN has multiple functions. In its membrane-bound form, APN is involved in complex functions in cells, such as peptide cleavage, immune cell chemotaxis, and monocyte cell adhesion^42, 43^. One of the best-studied characterizations of APN is its receptor function in CoVs, including TGEV, HCoV-229E, feline infectious peritonitis virus, canine CoV, and PRCV from the genus *Alphacoronavirus*^31, 35, 44, 45^. In this study, bestatin, an APN inhibitor that binds to its catalytic site, failed to affect PDCoV infection. However, two inhibitors that alter APN epitope conformation both decreased PDCoV infection in IPI-2I cells. These data suggest that a specific conformation of the pAPN epitopes, not its enzymatic activity, is required in PDCoV infection.

CoV S proteins contribute to the first step of viral infection and seem to be a vital determinant of host range and tissue tropism. Considering that pAPN is a functional receptor for TGEV, but not for PDCoV, we analyzed differences between PDCoV and TGEV S protein structures. In general, S protein S1 subunits, which contain two independent, functional subdomains S1-NTD and S1-CTD, mediate viral entry into cells during CoV infection. Although MHV S1-NTD is reported to recognize sugar receptors and a unique protein receptor carcinoembryonic antigen-related cell adhesion molecule 1^34^, protein receptor recognition for most CoVs depends on S1-CTD^46^. In particular, APN receptor in several alphacoronaviruses, such as TGEV and PRCV, interacts with S1-CTD^35^. Understanding the differences between S1-CTD structures of various CoVs is helpful to explain the role of pAPN in viral infection. Therefore, we analyzed the possible recognition of pAPN by PDCoV and TGEV S1-CTD. We found that the β1–β2 and β3–β4 turns in TGEV S1-CTD strongly supported its interaction with pAPN. However, for PDCoV S1-CTD, only residues at the top of the β1–β2 turn contacted pAPN. The markedly shorter β1–β2 and β3–β4 turns in PDCoV S1-CTD may cause insufficient contact with pAPN during viral attachment. These findings may help explain the limited role of pAPN in PDCoV infection. PDCoV can be detected in other tissues of infected pigs^1, 4, 10, 47^, suggesting that another, unidentified protein may contribute to the PDCoV recognition and entry processes. In addition, the S gene of CoV has been shaped by recombination and positive selection that may have led to changes in receptor-binding affinity^48, 49^. For example, isolated MERS-CoV strains from a recent outbreak in South Korea had point mutations in S1-CTD and showed decreased binding ability to the cellular receptor^50^. A highly neurotropic MHV JHMV strain infected host cells through a carcinoembryonic antigen-related cell adhesion molecule 1 receptor-independent manner^51^. However, whether the evolution of PDCoV S1-CTD is responsible for the shorter β1–β2 and β3–β4 turns and impaired ability to use pAPN requires further investigation. Moreover, the receptor-binding region of CTD is believed to bear mainly epitopes recognized by CoV neutralizing antibodies^35, 50, 52, 53^. Thus, the receptor-binding regions of CoVs are under selective pressure from the host immune system. We speculate that the shorter β1–β2 and β3–β4 turns in PDCoV S1-CTD compared with TGEV S1-CTD may be the result of positive immune pressure from the host. Conformational changes help PDCoV to evade host immune surveillance, while the ability of PDCoV to hijack pAPN may be impaired. These findings imply that a yet unidentified receptor may be utilized by PDCoV in the process of viral evolution and variation.

In this study, PDCoV exhibited a low level of infection in HeLa cells, a human cell line. Purified PDCoV S1-CTD binds to both human and porcine cells with high affinity^27^, suggesting that a co-receptor(s) exists on the surface of both human and porcine cells. We therefore evaluated the role of hAPN in PDCoV infection. PDCoV efficiently infected non-susceptible BHK-21 cells expressing hAPN by transient transfection, as demonstrated by detection of PDCoV S protein-specific fluorescence. However, TGEV failed to infect BHK-21 cells overexpressing hAPN (data not shown). These observations suggest that PDCoV may employ both pAPN and hAPN, while TGEV can only employ pAPN. In addition, a previous study suggested that calves are also susceptible to PDCoV infection^54^. Although no diarrhea or other clinical signs were observed in PDCoV-inoculated calves, persistent fecal viral RNA shedding and serum IgG antibody responses against PDCoV were detected^54^. These phenomena remind us of the potential risk for PDCoV infection with cross-species transmission.

In addition to the protein receptor that is responsible for CoV S1-CTD, host sugar receptors also interact with S1-NTD to facilitate initial viral attachment to cells in some alpha- and betacoronaviruses^55, 56^. The structural model of PDCoV S1-NTD presents a similar galectin fold to that of alpha- and betacoronaviruses, and the sugar-binding capability of PDCoV S1-NTD to mucin has been demonstrated through enzyme-linked immunosorbent assay^27^. The specific effects of sugar receptors in PDCoV infection deserve deeper study.

In summary, our results illustrate that pAPN could support PDCoV infection, independent of its enzymatic activity. However, whether pAPN promotes PDCoV infection through direct interaction with PDCoV S protein or with the cooperation of other host proteins must be further elucidated. The findings of slight infection in HeLa cells and successful infection in non-susceptible BHK-21 cells expressing hAPN suggest that a possible co-receptor exists in both porcine and human cells, revealing the potential risk for PDCoV cross-species transmission. Therefore, detailed study focused on virus-host interactions and identification of critical functional receptor usage is necessary to increase the understanding of PDCoV infection.

## Materials and methods

### Cells, viruses, and antibodies

IPI-2I cells (porcine intestinal epithelial cells), BHK-21 cells, and HeLa cells were obtained from the China Center for Type Culture Collection (Wuhan, China). LLC-PK1 cells were acquired from the American Type Culture Collection (ATCC CL-101; Manassas, VA, USA). IPI-2I, BHK-21, and LLC-PK1 cells were cultured in Dulbecco’s modified Eagle medium with 10% fetal bovine serum (Invitrogen, USA). HeLa cells were grown in RPMI 1640 medium with 10% fetal bovine serum. These cells were maintained in 5% CO_2_ at 37 °C. PDCoV strain CHN-HN-2014 (GenBank accession no. KT336560)^10^ and TGEV strain WH1 (GenBank accession no. HQ462571) were isolated in 2014 and 2010, respectively, in China. Mouse monoclonal antibodies against TGEV M, TGEV N or PDCoV S, PDCoV N were created in-house. APN antibody was purchased from ABclonal (China). An anti-Flag rabbit polyclonal antibody (MBL, Japan), Alexa Fluor 594-conjugated donkey anti-rabbit IgG (Santa Cruz, USA), and Alexa Fluor 488-conjugated donkey anti-mouse IgG (Santa Cruz, USA) were used for indirect IFA. Horseradish peroxidase-conjugated goat anti-mouse antibody (Beyotime, China) and horseradish peroxidase-conjugated goat anti-rabbit antibody (Beyotime, China) were applied in western blots.

### Construction of plasmids

pAPN and human APN (hAPN) eukaryotic expression plasmids were constructed by cloning pAPN or hAPN cDNA into vector pCAGGS-Flag with an N-terminal Flag tag. To knockout the pAPN gene in IPI-2I cells, two single-guide RNAs (sgRNAs) targeting exon 1 (5′-GGTAGGCGGTACCGGTTCCA-3′ and 5′-GTCTGTCTGTGGTGTACGCCC-3′) were designed and inserted into the PX459 background to generate sgRNA1 and sgRNA2 expression plasmids. All plasmids used were confirmed by sequencing.

### Treatment with APN-specific inhibitors or antibody

APN inhibitors bestatin (TargetMol, USA), 2,2′-dipyridyl, and 1,10-phenanthroline (Sigma, USA) were each dissolved in water at a concentration of 200 mM. The inhibitors were then diluted to various concentrations for use at 300 μM (bestatin), 250 μM (2,2′-dipyridyl), and 15 μM (1,10-phenanthroline) as previously described^36^. IPI-2I cells cultured in 24-well plates were pre-incubated with inhibitors, rabbit polyclonal antibody against APN, or anti-Flag rabbit polyclonal antibody (control) for 1 h and subsequently infected with PDCoV [multiplicity of infection (MOI) = 2] for 1 h. The cells were further washed three times with Dulbecco’s modified Eagle medium and maintained with inhibitors, APN antibody, or control antibody in cell culture containing 2.5 μg/ml trypsin. At 24 h post infection, cells were collected for IFA, and PDCoV titers in LLC-PK1 cells were determined by 50% tissue culture infective dose (TCID_50_).

### Internalization assay

IPI-2I-APN^WT^ and IPI-2I-APN^KO^ cells in 24-well plates with 90% confluence were inoculated with PDCoV (MOI = 30) for virus attachment at 4 °C. After 1 h, the infected cells were washed three times with cold PBS and cultured at 37 °C for another 1 h to allow virus internalization. Then, the infected cells were washed with citrate buffer solution (pH = 3) to remove the bound but non-internalized virus particles. The cells were harvested for RT-qPCR and western blot to evaluate the effect of pAPN on PDCoV internalization.

### Indirect IFA

IPI-2I cells, BHK-21 cells, or HeLa cells in 24-well plates were seeded on glass coverslips (NEST). Cells were washed three times with phosphate-buffered saline (PBS) and then sealed with 4% paraformaldehyde in methanol for 15 min and methanol for 15 min. Bovine serum albumin (5%) diluted in PBS was used to block cells for 1 h, and mouse monoclonal antibody against PDCoV S, TGEV M, or anti-Flag rabbit polyclonal antibody was then added and incubated for 1 h. After three washes with PBS, Alexa Fluor-conjugated secondary antibodies were added and incubated for 1 h, followed by 0.01% 4′,6-diamidino-2-phenylindole (DAPI) staining for 15 min to detect nuclei. After three washes with PBS, fluorescent images were examined by confocal laser scanning microscopy (LSM 510 Meta, Carl Zeiss).

### pAPN knockout by CRISPR/Cas9 genome-editing system

Knockout of the pAPN gene in IPI-2I cells was performed as previously described^57^. sgRNA1 and sgRNA2 expression plasmids targeting exon 1 of pAPN gene were constructed. The two sgRNA expression plasmids (each 0.5 µg) were mixed and transfected into IPI-2I cells cultured in 24-well plates. After 24 h, 1 µg/ml of puromycin (Sigma, USA) for IPI-2I cells was added to the cell culture for positive cell selection. After 48 h incubation, surviving cells were treated with trypsin and harvested to detect indel mutations by the surveyor nuclease assay. Clonal cell lines were isolated by dilution. Approximately 60 cells in 10 ml of Dulbecco’s modified Eagle medium were plated in each 96-well plate. pAPN knockout clonal cell lines were confirmed by sequencing and western blot.

### RNA extraction and RT-qPCR

Total RNA was extracted from PDCoV-infected cells by TRIzol reagent (Promega, USA). Using AMV reverse transcriptase (Takara, Japan), RNA (1 μg) was then reverse-transcribed into cDNA, which acted as the template in the SYBR Green PCR assay. RT-qPCR primers targeting PDCoV nsp16 (nsp16-F: 5′-GCCCTCGGTGGTTCTATCTT-3′, nsp16-R: 5′-TCCTTAGCTTGCCCCAAATA-3′) were used to measure PDCoV genome RNA copies.

### Western blot analyses

Cells cultured in six-well plates were harvested with lysis buffer (Beyotime, China) and boiled for 10 min with sample loading buffer (Beyotime, China). The samples were then resolved by sodium dodecyl sulfate-polyacrylamide gel electrophoresis, and proteins were transferred to polyvinylidene difluoride membranes (Millipore, USA). The membranes were blocked with 5% bovine serum albumin at room temperature for 2 h and incubated with rabbit polyclonal antibody against APN or mouse monoclonal antibody against PDCoV N or TGEV N for 3 h. After washing three times, the membranes were incubated with horseradish peroxidase-conjugated anti-polyclonal or -monoclonal antibody for another 1 h and washed three times. Proteins were detected using a western blot analysis system (Bio-Rad).

### Homology modeling

The structure information of proteins evaluated in our study was obtained from the Protein Data Bank (PDB) library. pAPN (PDB ID 4F5C), TGEV S (PDB ID 4F2M), and PDCoV S (PDB ID 6B7N) were chosen for analysis of S1-CTD and pAPN using PyMOL software (https://pymol.org/2/).

### Statistical analysis

All experiments were performed in triplicate. Data are shown as the mean ± standard deviation (SD). Student’s *t* test was used to measure significant differences between groups. *P* values < 0.05 were considered statistically significant.
